# Understanding of HIV/AIDS in the international border area, Manipur: Northeast India

**DOI:** 10.1017/S0950268818003564

**Published:** 2019-03-01

**Authors:** A. L. Sharma, T. R. Singh, L. S. Singh

**Affiliations:** Cancer and Molecular Biology Division, Department of Biotechnology, Manipur University, Canchipur-795003, Manipur, India

**Keywords:** AIDS, HIV/AIDS, HIV disease (AIDS), virology, virology (human) and epidemiology

## Abstract

Manipur, an international border region has the highest incidence of human immunodeficiency virus (HIV)-1 infection in India. Nevertheless, there have been no analytical reviews of research article published within this region. In this review, the authors aim to draw the attention of policy makers, medical practitioners and researchers in adopting new strategies to limit the expansion of HIV/acquired immunodeficiency syndrome (AIDS) not only in Manipur but also in other international border areas. A systematic search for published literature in last decade was performed based on the keywords ‘Manipur’ and ‘HIV’ using the PubMed. Twenty-six articles were selected and reviewed. There were high incidence of drug resistance (53%), emergence of recombinant virus (32%) and increased incidence of co-infection with hepatitis C virus. The prime cause of the HIV is due to the uses of ‘heroin’ smuggled from the ‘South Asia Golden Triangle’ and complex patterns of cross-border movement for trade and commerce. The drug abuse, social stigma, geographical location and resource limitation and socio-political problem of the region have contributed strongly on spreading and failure of preventively programme of HIV/AIDS. This review will provide vital knowledge for the policy makers and clinicians for sentinel surveillance of AIDS pandemic in Manipur and other international border regions.

## Introduction

Human immunodeficiency virus/acquired immunodeficiency syndrome (HIV/AIDS) is a life-threatening disease and remains an immense threat to all over the world [1]. Some countries are severely affected, of which India is one of them. India has the world's third-largest population suffering from HIV/AIDS, first and second being South Africa and Nigeria, respectively. HIV/AIDS remains a problem in India ever since it was detected for the first time in the year 1986 among the female sex workers (FSW) in Chennai [2]. It was assumed that foreigners in India were responsible for the HIV infections since they were travelling frequently in and out of the country. Sexual mode of HIV transmission was the main mode of HIV transmission in India. Initially, the progress of HIV epidemic was relatively slow but over the years the number of HIV-infected persons has increased and become a major public health problem. The spread of HIV in India was primarily restricted to the southern and north-eastern regions of the country.

Four years later, HIV case was registered for the first time in Manipur, an international border region to Myanmar, from a blood sample of an intravenous drug user (IDU) [3]. Since then, Manipur, a total area of about 22 327 km^2^ with a population of 2.7 million (density of 120 people per square kilometres) is reported to be hard hit epicentres of HIV/AIDS epidemic in India. Manipur, a far north-eastern corner of the country with hardly 0.2% of country's population is contributing nearly 8% of India's total HIV-positive cases. HIV infection rate among the IDUs had increased tremendously from 2% to 3% in 1989 to >50% in 1991 and 64% in 2000 [4]. The severity linked with the HIV/AIDS pandemic remains a problem to the state. HIV/AIDS has emerged as a serious public health emergency and seems like impossible task to halt the ever-increasing rate. Unlike other states of India, HIV transmission in this region is mainly correlated with the sharing of HIV-infected injecting equipment/needles among the IDUs [5]. IDUs are the easy prey for contracting HIV. Although the epidemic was initially described among IDUs, HIV is no longer confined to IDUs but it begins to appear in the general public.

In this review, the authors examine the causes, challenges and current scenario of HIV/AIDS in Manipur to draw the attention of policy makers, medical practitioners and researchers in adopting new strategies to limit the expansion of this deadly disease.

## Material and methods

This scoping review was determined based on the following frame works; identifying the research publications; sorting out the relevant studies; opting the studies for review; gathering the information; summarizing and reporting [6]. The review of published literature was deemed the most apt methodology for this project due to the lack of analytical reviews on HIV/AIDS in this international border area. We have tried to identify, organise the common themes and gaps regarding the transmission, molecular epidemiology, drug resistance (DR) and co-infection. This review was undertaken to examine the extent, range and nature of research activity; to determine whether a review is feasible and of value, to summarise research findings to policy makers, medical practitioners and identifying gaps in the existing evidence.

In August 2018, based on the keywords ‘Manipur’ and ‘HIV’, an advanced systematic search for published literature in English in the last decade (since 2008) was performed using the PubMed (https://www.ncbi.nlm.nih.gov/pubmed), a free search engine accessing primarily the MEDLINE database of references and abstracts on life sciences and biomedical topics. Only the publications from 1 January 2008 to 31 August 2018 were taken into consideration. All the displayed articles were accessed and identified. The articles were further accessed in ISI Web of Science database by Clarivate Analytics. Only those publications in the journals included in the Journal Citation Reports (JCR) list, 2018 (Clarivate Analytics/Thomson Reuters) were selected for this study. The selection of the article based on the journals within the ISI Web of Science database is to maintain a quality or standard of article to be reviewed in this study. The title, abstract, journals and full text of eligible publications were scrutinised independently by two authors. The articles which report empirical data with respect to the transmission, molecular epidemiology, DR and co-infection in Manipur were further considered. This systematic search clearly indicated that the review literature on this topic is limited. We further access the electronic databases at the Manipur AIDS Control Society (MACS) (https://manipursacs.nic.in/), Manipur for additional information. The final step in our methodological approach involved plotting the information from published literature; extraction of relevant data and charted them to assist with our analysis of the findings.

## Results

**Fig. 1.**
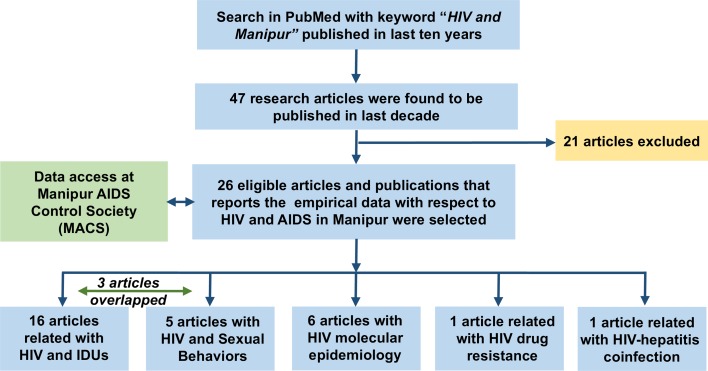
A flow chart showing the process of articles selection for this review.

Forty-seven articles were found to be published since a decade when we search in the PubMed using the keyword mentioned above. Out of the 47 articles, 26 articles were selected for this study. The selection was based on the articles that published in the journal within the web of science database and also related to empirical data with respect to transmission, molecular epidemiology, DR and co-infection of HIV/AIDS in Manipur. Three articles were found to be overlapped with the HIV literatures that described both the IDUs and sexual behaviour. We have also included the data from MACS that focused on the demographic data, socio-economic data, incidence rate, etc. The articles have been further classified as shown in Figure 1.

### HIV and injecting drug users

Injecting drug use has received increased attention because of the high risk of HIV transmission and, more recently, hepatitis B virus (HBV) and hepatitis C virus (HCV). Since last decade, there were at least 16 publications related with IDUs. In 2008, Mahanta *et al.* [7] showed that 98% of the IDU use injecting ‘heroin’ and 63% of them shared their injecting equipment and containers. This unsafe injecting practice exposed themselves in the risk of contracting blood-borne viruses. According to Kermode *et al.* [8] in 2009, the main theme of using drugs was for pleasure-seeking, influence of peers and economic reasons. The idea of injecting drug was initiated by another person (most commonly a friend, who helps in injecting the drugs) in the well-established social networks. Another study showed that to deal the problem of IDUs, opioid substitution therapy is effective and leads to the improvements in the quality of their lives [9, 10]. A study by Armstrong *et al.* [11] in 2011 showed that those IDUs having the knowledge of HIV prevention services are more likely to engage in safe injecting, sexual practices and avoid risky characters. Chakrapani *et al.* [12] in 2011 showed that effective HIV prevention and care programmes for IDUs may hinge on several contexts; supportive government policy on harm reduction programmes; an end to harassment by military, and anti-drug groups, with education of these entities regarding harm reduction and creation of partnerships with the public health sector. However, in another study by Suohu *et al.* [13] in 2012 revealed that if the IDUs practice the unsafe injection by sharing injecting equipment and risky sexual practices, it magnifies the risk of HIV infection. Medhi *et al.* [14] in 2012 estimated the sizes of IDU population in five districts of Manipur. Not only men but women also used injecting heroin according to Kermode *et al.* in 2012 [15]. The study further showed that heroin on women has negative health impact such as reproductive health, mental health, social exclusion, violence, children's welfare and financial difficulties. Lalmuanpuii *et al.* [16] in 2013 showed that harm reduction approach in Manipur could be even managed better. In 2013, Kermode *et al.* revealed that 64% of young women (mean age of 31 year) who used heroin and alcohol were widowed or divorced. It also further indicates that women used drug and alcohol to avoid symptoms of withdrawal, to suppress emotional pain, to overcome the shame of sex work, pleasure and widowhood [17]. Another study by Armstrong *et al.* [18] in 2014 identified that the risky injecting practices were more common among younger people. Goswami *et al.* stated that improvements in safe injection practices and consistent condom use with regular partners enhance the HIV preventives measure [19]. Armstrong *et al.* [20] in 2014 showed that most of the HIV-positive people who inject drug in Manipur were not aware of their HIV status and practice unsafe injection and sexual activities. However, when they become aware of their HIV status, they avoid their high-risk behaviours. So, it is utmost important to heighten the HIV testing coverage scale across the state. However, Ganju *et al.* [21] in 2016 revealed that HIV testing among IDUs is low. Urgent program to build awareness among the IDUs about their HIV status is necessary to limit the expansion of HIV, timely treatment and care. Phukan *et al.* [22] in 2017 provided a useful program to understanding the network pattern of injecting drug users for enriching the HIV prevention in this region.

### HIV and sexual behaviours

In the last 10 years, there were five publications related with HIV and risky sexual behaviours. In 2008, Mahanta *et al.* [7] showed that nearly half of IDUs have engaged in sexual activity with at least one female in past one year. In another study by Suohu *et al.* [13] in 2012 indicate that a significant proportion of IDUs engage in unsafe sex and have multiple sexual partners. They have more sexual partners as compared with non-injecting heroin users. More than 27% IDUs reported having had unsafe sex with two or more female partners within a year. These provide a higher chance of transmitting or contracting HIV. Armstrong *et al.* [18] in 2014 also showed that injecting drugs user aged 18–24 years had two or more female sexual partners (50.2%) compared with aged 35 or older (10.9%) indicating youth have higher risk. In the same year, Mishra *et al.* suggested that 40% of IDUs had a casual sexual partner and among those who had casual sexual partners, 65% of them have inconsistently used condom [23]. IDUs who shared needles/syringes were more likely to engage in unprotected sex with their regular partners. Similarly, IDUs who reported inconsistent condom use with casual partners were more likely to report unprotected sex with their regular partners. Kermode *et al.* [24] in 2015 reported that condom use with regular partners was poor: 40.6% used a condom the last time they had sex with their regular partners, and only 10.7% reported consistent condom use with their regular partners. Many participants with regular partners (40.2%) had more than one sexual partner in the last year, 29.5% had casual sex and 6.1% paid for sex in the last year. Consistent condom use with casual sexual partners was 32.9% and with paid partners was 36.5%. Half of those with regular partners (51.0%) had never had an HIV test, and 14.3% were HIV-positive.

### Molecular epidemiology

During the last 10 years, there were six publications related with molecular epidemiology of HIV as shown in Table 1. In the year 2009, a study by Sarkar *et al.* on 30 HIV-infected IDU samples recruited from Manipur suggested the possibility of emerging new HIV-1 strains along with the dual infection [25]. The prevalence of subtype C with BC recombinants in the HIV tat gene was shown by Mullick *et al.* in year 2010 [26]. In the same year, a study was conducted on HIV infection and host genetic mutation from the IDU samples of north-eastern states of India, including Manipur. It revealed that the absence of CCR5 mutant gene which suggests that they do not have any additional protection against HIV infection. They were vulnerable to acquire HIV infection due to high-risk behaviour and other related factors [27]. Sarkar *et al.* in two independent studies in year 2012 revealed the emergence of unique recombinant forms (URFs) [28, 29]. Later Sharma *et al.* further analysed the viral subtype using larger sample size based on two gene pol and/or tat-vpu-env gene [30], it showed that the HIV-1 genotypes distribution was 65.45% (72/110) subtype C, 32.73% (36/110) URFs and 1.82% (2/110) subtype B. The distribution of HIV-1 genotypes among the risk groups was: heterosexual: 58.33% (35/60) subtype C, 38.33% (23/60) URFs and 3.34% (2/60) subtype B; IDUs: 85.36% (35/41) subtype C, 9.76% (4/41) URFs and 4.88% (2/41) subtype B; mother to child (MTC): 50% (3/6) URFs and 50% (3/6) subtype C and blood transfusion: 100% (3/3) subtype C.

**Table 1.** Molecular epidemiology of HIV/AIDS in Manipur, North-east India: articles published since a decade and identified within the review

Sl.no	Analysis	Results	Year of study	Reference
1	Multiregion hybridisation assay (30 samples)	15 Subtype C, 1 subtype B, 14 with dual and multi-genomic recombination pattern	2009	Sarkar *et al*. [25]
2	Sequencing of *tat* gene (15 samples)	Prevalence of subtype C with few BC recombinants for the tat gene	2010	Mullick *et al*. [26]
3	PCR and restriction digestion (306 samples)	Manipur population do not have any additional protection against HIV infection	2010	Sarkar *et al*. [27]
4	Sequencing (1 sample)	Mosaic structure of subtype C back bone with Subtype B	2012	Sarkar *et al*. [28]
5	Sequencing (1 sample)	Subtype C backbone with India, while the subtype B insertions resemblance with Thailand.	2012	Sarkar *et al*. [28]
6	Sequencing (130 samples)	65.45% subtype C, 32.73% URFs and 1.82% subtype B	2016	Sharma *et al*. [31]

### Drug resistance

In the last decade, there was only one publication related to resistance to HIV drugs. There has been no report on HIV DR profile in the north-eastern region of India till our recent study in the year 2016. According to our recent studies, 53% of HIV-infected antiretroviral therapy (ART) experienced individuals in Manipur harbour DR mutation at different DR sites [31]. It also further revealed that 29%, 37% and 8% have mutations at the target sites of nucleoside reverse transcriptase inhibitors (NRTIs), non-nucleoside reverse transcriptase inhibitors (NNRTIs) and protease inhibitors (PIs) sites respectively. Predominant drug-resistant mutations at reverse transcriptase (RT) genes were M184V, T215Y, M41L and V108I and H221Y while at protease (PR) genes were M46I and I47V. Among the high-risk groups, IDUs have the highest number of drug-resistant mutations followed by heterosexual individuals. It was further shown that drug-resistant mutations at the target sites of RT inhibitors are high and these were found to have developed resistance to the primary ART drugs that are used in Manipur.

### Co-infections

Since 2008, there was a single publication related with co-infection of HIV with other viruses. Kermode *et al.* [32] in 2016 particularly focus on IDUs residing in two districts of Manipur. Among the 31% of HIV-positive IDUs, 95% were co-infected. HCV infection was associated with district, longer duration of injecting, injecting at least once daily, generally injecting with a used needle and syringe, and having had an HIV test. HCV/HIV co-infection was associated with district, older age, being employed, being widowed/divorced, longer duration of injecting and feeling at risk of HIV infection. The study also showed that high prevalence of HCV among the people living with IDU in Manipur where prevention, diagnosis and treatment options are limited.

## Discussions

**Fig. 2.**
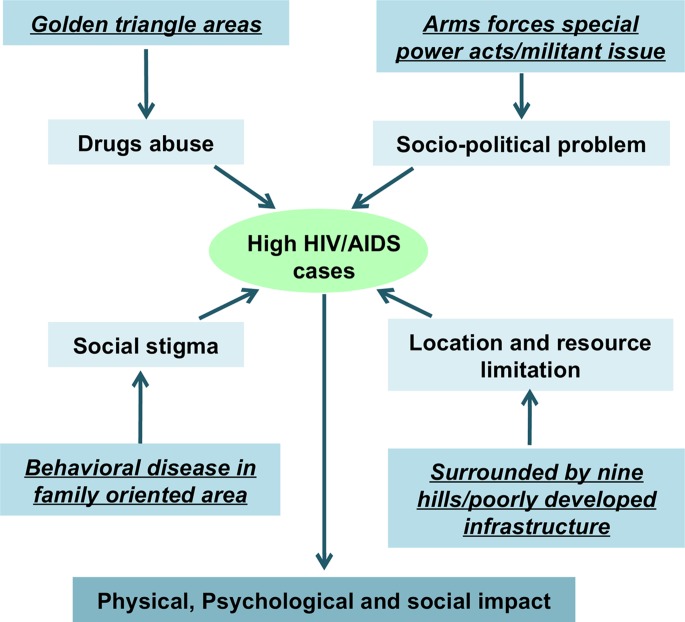
Pictorial representation of factors which influence high HIV/AIDS.

This review article provided an opportunity to investigate and update the HIV/AIDS epidemics in the Indo-Myanmar border. It is conducted based on the literature published with relevant data of HIV since the last decade. In this study, the authors show the factors which influenced high case of HIV/AIDS in Indo-Myanmar international border. The entire international borders all over the world have more or less similar issue. Behavioural networks also exist on the other segments of the border which causes havoc not only to both the countries but also to all the neighbouring countries. Based on the literature published, the authors show that drug abuse, social stigma, geographical location and resource limitation and socio-political problem of the region have contributed strongly on spreading and failure of preventive program of HIV/AIDS (Fig. 2). These factors are not just confined to this Indo-Myanmar international border location.

Drug users and addiction have been intimately linked with HIV/AIDS since the beginning of the epidemic. Among the drug users in Manipur, ‘heroin’ which is locally better known as ‘number four’ is the most commonly (98%) used injecting drug [33]. Ever since, the emergence of ‘heroin’ in the year 1982 from South Asia Golden Triangle, the availability in illegal drug market has sharply increased. The route of illegal drug trafficking is assumed to be via twin town Tamu (Myanmar) and Moreh (Manipur, India). Moreh (Tengnoupal district of Manipur, India) is an important town in connection with the India–Myanmar relationship. It is one of the developing places in the field of trade and commerce on the Indian border with Myanmar while the city of Tamu on the other side of the border. Due to aforementioned reasons, the contiguity to the Golden Triangle has been the most important reason for the rapid rise of IDUs [34]. Some youth also use oral pain reliever drugs in combination with heroin as leisure-seeking drugs [35]. Both forms of drugs can have serious negative health effects. However, use of ‘heroin’ intravenously is more deadly as it spreads the blood-borne viruses like HIV, HBV and HCV through shared injecting equipment [36]. Young (aged 18–24 years) IDUs shared the injecting equipment more often when compared with the IDUs aged more than 35 years [18]. The teenager and middle-aged people (mixed age group peoples) of rich families use the drug as a kind of fashion initially. Economically middle and low class originated youths follow the trend. Addicted youths afford the expenditure on drugs by stealing or extorting small to valuable goods from others or their own houses. The crimes related to drugs addiction increases day by day and therefore such crimes have become a social problem. Domestic chaos and violence to women and children were the ultimate impact of drug users [37]. The situation has become exacerbate because of wrong belief that drug-addicted youth give up addiction when they have their own family responsibility, and therefore parent insist the addicted youth for marriage without knowing of the fact that the drug-addicted youth might have been infected with HIV or hepatitis (HBVs/HCVs). It is worth to mention that more than half of the IDUs were infected with HIV, while substantial proportion of HIV-positive people with injecting drug users were still unaware of HIV-positive status [20]. Therefore, HIV-infected men have the risk of contracting of HIV infection to their female sexual partner [38]. These created a chance of transmitting virus knowingly or unknowingly to spouses and then to their children [39]. It was previously reported that 95% who shared injecting equipment were engaged in unprotected sex with regular sexual partners, while 97% of IDUs who inconsistently use condom with casual partners were engaged in unprotected sex with their regular partners [23]. Earlier, HIV/AIDS cases were confined among the IDUs. However, HIV/AIDS epidemic has now penetrated in the general population through the risk behaviours of IDUs. Meanwhile, housewives with single partners are gradually accounting for a larger proportion of infections. These monogamous women are primarily put at risk by the IDUs of their husband, from whom their infection is most probably acquired. The housewife is becoming the new face of the epidemic. However, when IDUs become aware of their HIV status, they reduce their high-risk behaviours. So, it is utmost important to heighten the HIV testing coverage scale across the entire international region.

FSWs are also an obvious risk for sexual transmission of HIV and hepatitis virus. Some female drug users engage in sex work to support their drug and alcohol use and *vice versa* [15]. Since HIV/AIDS is mainly associated with the behavioural nature of a person, many people hesitate to disclose their HIV status [41]. There have been various reports of HIV/AIDS-infected persons being discriminated, stigmatised and ostracised by society. The widowers of HIV/AIDS spouses are not accepted by even own family [42]. Therefore, stigma creates isolation mentally as well as physically from own family, friends and community resulting into depression or sometime suicide [43]. The problem has wrought havoc in the state and it is the women who have become the casualties. Thus, stigma forms a barrier for effective HIV care and treatment [44]. Even though antiretroviral medications are available for free, many individuals in the state choose not to seek treatment because of stigma and discrimination. Even though ART drug regimes had been started since in 1 April 2004 in Manipur, it remained inaccessible among HIV-infected patients due to hesitation on being registered themselves in ART Centers. This raises a serious public health issue for diagnosis and ART treatment. This study also indicates that the problems posed by injection drug use and HIV infection are particularly challenging in border regions.

Due to the geographical location and lack of good HIV/AIDS surveillance despite being high prevalence, the state, Manipur is deeply affected economically and socially due to the HIV epidemic. There are many HIV viral load test (VLT) centres in the country to monitor the progression of the disease, but none in the state has the highest HIV adult prevalence in the country. Therefore, blood samples are collected from HIV-positive patients and sent outside the state for VLT. Since blood samples are transported to the viral load testing centres only in bulk by air or surface transportation, blood samples are stored until sample collection reaches certain numbers. Therefore, patients get their viral load result after 3–4 weeks and by the time doctors know viral load of a patient, there is always possibility that the viral load has increased many folds. It may be another contributing factor of failure ART. In such situation, lives of HIV-positive patients are in danger since viral load is vital for different ART treatments. This shows that the necessity for effective HIV/AIDS surveillance even in resource-limiting area.

The frequent political and social turmoil in the state worsens the HIV/AIDS situation. The insurgency movements of Manipur led to enforcement of strict law by government in the form of Armed Forces Special Powers Acts (AFSPA). Under this act, possession of any illegal or suspicious goods is liable to be sent to custody without any warrant. Therefore, armed forces and local police are given special power to arrest any suspect including IDUs. The above situations consequence the ineffectiveness of the syringes exchange program, initiated to prevent the spread of HIV through sharing of syringes among IDUs in this region since carrying syringes is risky. As such preventive measure fails to stop sharing of injecting equipment. Hence, the law and order situation also influence negatively on the adherence to drug since lifesaving drugs could not be available in time due to sudden curfew and strikes. The socio-political problem hinders the effective programs initiated to limit the HIV transmission. The above factors finally influence the molecular epidemiology, DR and coinfection with other types of virus.

The prevalence of HIV-1 subtypes varies greatly depending on the geographic region [45]. The subtype C is the most prevalent among all HIV-1 genetic subtypes and is predominant in India [46]. However, recombinant form of virus is most visible in border areas where different genetic variety meets. The extensive variability of HIV-1 genetic forms complicates diagnosis, treatment and prevention of HIV-1 infection. Therefore, HIV-1 subtype characterisation is becoming an important aspect to adequate clinical management of HIV-1-infection. Mullick [26] in 2009 identified BC recombinant based on the tat gene and long terminal repeats from IDUs of Manipur. However, we have conducted extensive analysis of molecular epidemiology of HIV in the year 2016 using large sample size [30]. Surprisingly, our study revealed the emergence of URF (32%) (Table 2). Manipur being a state of transit and commercial area that trade and connect the south East Asia may have the potential to introduce the complex genetic variant. The impact of foreign travellers and immigrants due to cross border transport in HIV epidemic is unknown. However, to identify the URFs found were formed locally or derived from Myanmar and/or other border area, molecular epidemiology across the border area need further investigation.

**Table 2.** Fact files of HIV/AIDS in Manipur

Manipur		
Details	Data	Sources
State population (Census 2011)	2 855 794	Census 2011, Manipur
Virology	HIV-1 and HIV-2	MACS and Sharma *et al.* [40]
HIV-1 subtype (in 2016)	66% Subtype C 32% URF and 2% subtype B	Sharma *et al*. [31]
Prevalence of DRMs % (year)	53%	Sharma *et al*. [31]
First case reported	February 1990	MACS
Highest prevalence % (year)	16.72% (1996)	MACS
Risk groups	IDU, heterosexual	MACS
Control program (year established)	3 October 1996 (first in country)	MACS
Adult (15–49) prevalence (%)	1.15 (0.88–1.55)	NACO HIV estimates 2015
People living with HIV/AIDS	24 457 (18 748–33 362)	NACO HIV estimates 2015
Annual new infection	429	NACO HIV estimates 2015
AIDS related death	1146	NACO HIV estimates 2015
Need for ART	15 510	NACO HIV estimates 2015

The ART has greatly advanced the management of HIV-1 infection [47]. ART drugs targeting the viral RT and PR activities can suppress HIV-1 replication to undetectable levels, leading to significant clinical benefit [47]. However, the main cause for the failure of ART is the presence of drug-resistance-associated mutations in the polymerase gene of HIV-1 [48]. The emerging of HIVDR is mainly due to drug pressure of ART. The drug-resistant mutated virus can be transmitted from one individual to another. The effect of transmitted DR is a cause of concern for the scaling up of HIV programs, as HIV resistance also affects the efficacy of MTCT prophylaxis. There has been no report on HIV DR profile in the north eastern region of India till our recent studies. According to our recent studies, 53% of HIV-infected ART exposed individuals in Manipur harbour DR mutation at different DR sites. The study revealed that 29%, 37% and 8% have mutations at the target sites of NRTIs, NNRTIs and PIs sites, respectively [34]. Individuals with drug-resistant mutation represent a great challenge for the future ART program. There is a necessity for monitoring the effectiveness of ART program to enhance the appropriate treatment responses.

HIV and HBVs/HCVs, the three important blood-borne infectious diseases, are the major public health concerns [49]. Due to co-infection, HIV and HBV/HCV simultaneously interact in a host complicating pathogenesis and disease progression of these two infections [50]. The deleterious effect of HIV leads to more rapid progression towards end-stage liver diseases (liver cirrhosis and hepatocellular carcinoma) and higher risk for liver disease-related mortality in HIV/HBV co-infected individuals as compared with those infected with HIV or HBV only. Since the IDU is the chief transmission route of HIV/HBV co-infection in Manipur, the vulnerability of co-infection is predominant. HIV-HBV co-infection had 17 times higher incidence of liver disease-related deaths than HBV mono-infected ones. There are limited molecular studies on co-infection of HIV-HBV-HCV in the north-eastern region of India. However, Kermode *et al*. [32] in 2016 particularly focus on IDUs residing in two districts of Manipur. Among the 31% HIV-positive IDUs, 95% were co-infected. Co-infection with other virus HBV/HCVs disease hampers the response to treatment [51]. This shows the high rate of co-infection with other blood-borne virus in this region. There is an urgency to manage the co-infection to limit the expansion of viral-borne diseases. Further, more research is required on the social, economic and cultural determinants of HIV/AIDS diagnosis, treatment and response to ART program. The collaborative approaches should be initiated so as to raise awareness and discuss risks associated with blood-borne viruses. Entire international border areas need to be enhanced the screening and detection systems of HIV and other blood borne viral diseases.

## Conclusion

Since Manipur has the highest HIV prevalence rate in the country, any study with respect to HIV/AIDS in this region need to encourage not only to solve the health issues, but also the development issue of the country as whole. The prime cause of the HIV pandemic in this international border area is the uses of ‘heroin’ which are smuggled from the ‘South Asia Golden Triangle’. This study indicates that drug abuse, social stigma, geographical location and resource limitation and socio-political problem of the region have contributed strongly on spreading and failure of preventive program of HIV/AIDS. The HIV pandemic in this border region is much more complex than any other region of the country due to the emergence of recombinant HIV forms and DR HIV-1. Intravenous drug use and complex patterns of cross-border movement have combined to produce serious problems of HIV/AIDS pandemic. Improved knowledge about the causes, challenges and current scenario of HIV/AIDS should lead to improvement in the investigation and treatment of patients. However, in-depth studies such as the role of the emerging genetic variant in response to antiretroviral treatment, effect of co-infections with different viruses, etc., need to be inspired in all international borders to limit the expansion of more complex form of virus. It has also become evident that the importance to heighten the HIV testing coverage scale across the entire international region to reduce the risky behaviour of IDUs. The collaborative approaches to HIV prevention across all international borders as whole are anticipated in future. These will not only improve HIV prevention in international border region but also help to increase and diversify cross-border collaboration in HIV prevention and public health. These will certainly help to thrust for positive changes in governmental policies related to drug use and HIV prevention. Nevertheless, the information generated from this study will provide vital information for the policy makers, medical practitioners and researchers in adopting new strategies in restraining the transmission across the border area. The understanding developed from this study on the profile of the HIV-1 variants, DR, co-infections and main mode of transmission in a ‘geographic recombination hotspots’ may help to fight back the deadly AIDS pandemic.
